# Responsible Communication of Romanian Companies for Ensuring Public Health in a COVID-19 Pandemic Context

**DOI:** 10.3390/ijerph17228526

**Published:** 2020-11-17

**Authors:** Camelia-Daniela Hategan, Ruxandra-Ioana Curea-Pitorac, Vasile-Petru Hategan

**Affiliations:** 1 Department of Accounting and Audit, Faculty of Economics and Business Administration, West University of Timisoara, 16 Pestalozzi Street, 300115 Timisoara, Romania; camelia.hategan@e-uvt.ro; 2 Department of Economics and Economic Modeling, Faculty of Economics and Business Administration, West University of Timisoara, 16 Pestalozzi Street, 300115 Timisoara, Romania; 3 Institute for Social and Political Research, Faculty of Political Sciences, Philosophy and Communication Studies, West University of Timisoara, 4 V. Parvan Blvd, 300223 Timisoara, Romania; vasile.hategan@e-uvt.ro

**Keywords:** communication, public reports, risk, governance, investor relation, crisis, COVID-19

## Abstract

The COVID-19 pandemic has forced companies to respond to the threat of this risk and innovate in corporate governance. In order to reduce the risk of illness, one of the most applied measures by all companies was social distancing, but to avoid human interaction, companies had to adapt their communication strategies. The objective of the paper is to assess the risk management of Romanian-listed companies associated with COVID-19 focusing on their business communication with shareholders and stakeholders. To emphasis the communication we have chosen to analyze all public reports during the state of emergency of the companies listed on the main market at the Bucharest Stock Exchange. The empirical analysis consists of a panel data econometric model using maximum likelihood random-effects regression and a logistical regression to highlight the correlations between the dependent variables Public Reports and Business Continuity Plan and the analyzed independent variables. The study showed that in most cases, the companies had at least one public report, especially the one related to the annual shareholders meeting, a percentage of 21% of companies had two public reports, and only 17% of companies have published three or more reports. The companies that communicated the most were the ones belonging to the premium trading category, and the number of published reports was influenced by the communication evaluation indicator, profitability and by the announcement of the donations made.

## 1. Introduction

Under normal conditions, organizations provide information to other organizations, such as business partners, public administrations, credit institutions. This external communication aims to facilitate relations with stakeholders and increase trust in them. In times of crisis, corporate governance is faced with additional tasks, to establish new strategies and to have more interactions with its management. The declaration by the World Health Organization [1] of the COVID-19 pandemic on 11 March 2020 had a major impact on all areas of activities and on people’s lives.

Within the Sustainable Development Goals (SDGs), the United Nations has identified poverty and access to healthcare as problems, so COVID-19 pandemic is a challenge to change business models towards sustainability through public health measures and plans.

In order to reduce the effects of the COVID-19 pandemic and ensuring the public health of the people, the Romanian President declared a state of emergency for 60 days, from 16 March to 14 May 2020 [2,3]. For reducing the risk of illness, one of the most applied measures by all companies was social distancing because the healthcare system was taken unprepared in many countries, especially in Romania where there are many problems remaining from the communist period [4].

The objective of the paper is to assess the risk management associated with COVID-19 of Romanian = listed companies focusing on their business communication with shareholders and stakeholders. In order to achieve our objective, an analysis of the level of the communication, during the state of emergency, of the companies listed on the main market at the Bucharest Stock Exchange (BVB) was conducted. According to the data published by BVB at that time, 83 companies were listed, and the information about their public reports was taken from the Issuer Reporting Informational System (IRIS) platform of the BVB.

The methodology applied for the empirical analysis consists of a panel data econometric model using maximum likelihood random effects regression and a logistical regression to estimate the correlations between the dependent variables Public Reports and Business Continuity Plan and the independent variables: Vektor index, Profitability, Donations, Number of Employees, Trading Category and the form of ownership of Shareholders.

The study will contribute to the literature as it provides an analysis of the available data on the communication forms of listed Romanian companies during the period of crisis, highlighting the importance of communication with stakeholders. Because of the continuing pandemic crisis, many companies have encountered difficulties, such as layoffs, technical unemployment or even the collapse of their business and other financial problems, which require a stronger communication and expanding social responsibility actions, based in particular on environmental and social pillars [5].

The paper is structured as follows: the next section contains a brief of the relevant literature on corporate communication, while the third section describes the methodology of the research. The fourth section presents the results of the analysis and discussions. The last section includes the conclusions of the paper, limits and future research directions.

## 2. Literature Review

### 2.1. Background Regarding Responsibility and Business Ethics

The global pandemic has brought many challenges to the companies’ leadership, whose organizations have been subjected to unprecedented medical restrictions. This required a rapid adaptation of production strategies and rethinking flows to meet the requirements of protection for staff and the community where they activate. The actions of the managers were changed by quickly taking radical and responsible measures to ensure the protection of the company’s members but also for the rest of the community, and the society in general. Prior to the crisis, leadership policies were aimed primarily at the personal development of the management team, in order to achieve the set performance targets and to reflected the organization’s social responsibility measures. During the pandemic crisis, priorities have changed to develop organizational and social leadership, in an attempt to promptly respond to all the challenges.

The need to develop social leadership was previously anticipated in a dialogue by the economist Sander Tideman with the Dalai Lama, pointing to the need to implement social leadership in the business environment, as it “ensures a sustainable profit at the highest level of complexity that includes economic, social and ecological profit” [6]. This action can be activated in order to achieve the common goal pursued, to successfully overcome the state of pandemic crisis of humanity.

Taking explicitly the communication ethics developed in the field of public communication [7] the research we refer to concludes the need to develop ethical norms oriented towards transparency and accountability to be correlated with business ethics [8] oriented towards people, environment and life in general. This can become a transversal principle that will be applied by organizations, in the sense that these orientations become adjacent to the main economic activity, the production or the services. Another opinion [9] on the need for applied ethics in economics highlights the importance of the existence of a permanent dialogue between ethics and micro/macro economics, which must become a complex and necessary relationship between them.

The topic of moral responsibility applied in the context of business ethics was analyzed by a group of Romanian researchers [10], highlighting the philosophical foundations of responsibility. They referred to ancient philosophers who initiated the first concepts of morality, and next to the writings of Immanuel Kant, with his approaches to the subject of moral responsibility, associated with the concept of the person’s freedom. The analysis refers to the “heuristic of fear” derived from the work of a contemporary philosopher [11] who establishes the ethics of responsibility as one based on the concepts of prudence and fear, which is why this relationship between fear and responsibility is considered one of a positive nature [12]. As fear can also be associated with hope, not only with fear seen as a human weakness in the face of a crisis, and responsibility thus becomes the solution to overcome it.

The philosophical theories have been concerned with the limits of responsibility, where Jonas [11] saw responsibility as a necessary sacrifice to be made today for the good of future generations, other authors [12] regarded the limits of responsibility as excessive for the concept of responsibility, presenting the two directions of the effects of responsibility, for future generations and for the present generation. The same authors [12] analyzed the concept of responsibility applied to the business environment, wondering if the organization should be seen individually as a person, thus highlighting the transition through social responsibility to a new model of organization called “citizen enterprise”.

The restrictive measures generated by the pandemic crisis have accelerated and amplified some specific measures in the field of corporate social responsibility, to the detriment of achieving economic and market indicators, constantly monitored by leadership. This confirms other previous claims [13], that responsibility is seen as a duty or obligation to the community, but also as a consequence of a certain action, reaching the concept of collective responsibility, generating group solidarity, to act united in the context of the pandemic crisis, to overcome and remove the negative effects produced by it.

The conclusions of the Romanian researchers on business ethics [10] highlight a new ethical dimension of approaching organizational responsibility, seen “as a duty, obligation or self-obligation, both of the entrepreneur and of the company, and as a responsibility of the group or collective, and as a responsibility of the organization” which highlights the characteristics of a “moral organizational man ”, generating confidence in the actions taken in this field of Corporate Social Responsibility (CSR).

### 2.2. Corporate Communication and Investor Relations

Lately, the managers have become more motivated to use certain communication strategies to show that they fulfil the norms and expectations of the company where they operate [14,15,16,17,18,19]. An organization’s reputation develops as a result of the interactions and communications that take place between stakeholders and managers, representing a valuable resource for the company [20,21].

To avoid human interaction, companies had to adapt their communication strategies as part of the risk management associated with COVID-19. The capital market had a quick response to this announcement, the sharp drop in stock prices, but as the COVID-19 pandemic evolved, risks arose at the level of listed companies. In order to reduce the reputational risk of the companies, better communication with the shareholders and with the other interested parties is required. Hoffmann et al. [22] conducted a review of research on investor relations and found that after the onset of the financial crisis in 2008, the number of researches increased, which shows that periods of crisis are sources of data for researchers [23].

The impact of COVID-19 on stock markets has been studied by researchers since the beginning of the pandemic and in recent months many studies have been published that show the financial impact on investors and the impact on business continuity of listed companies from all over the world [24,25,26,27,28,29]. Thus, companies have to consider a number of new factors that affect their normal activity, such as the need for a Business continuity plan, also considering the possible psychological effects of the current situation on the workforce [30] or solutions to new types of debt and disputes that may arise [31]. A major impact on companies was the organization of general meetings of shareholders that needed to move to virtual [32] which led to changes in company laws [33].

Maldin-Morgenthau et al. [34] defined the steps that corporations must take during public health emergencies, such as pandemics, by establishing emergency response plans and clear channels of communication. Communication in times of crisis brings challenges to companies in the ability to seek new tools to communicate with investors [35] and to develop crisis management plans [36]. The reporting of financial indicators of companies, as a mechanism of corporate communication [37] was affected by the behavior of investors in crisis situations [38]. To counteract the negative effects, companies have adapted their strategy of communicating with investors [39] by officially publishing reports on the impact of COVID-19 on business and by posting on their website.

Lopatta et al. [40] conducted a study of 300 international companies on reporting practices during COVID-19 and found that firms that report on the impact of the pandemic in their annual report show a significant improvement in their returns compared to those which do not report. Thus, they showed that companies’ reporting practices play an important role in better understanding the reactions of current capital markets to the ongoing coronavirus pandemic. Thus, companies need to build a corporate governance policy that focuses on sustainability, well-being and IT infrastructure to meet any future challenges arising from the pandemic crisis [41].

Companies’ reporting practices are assessed by capital market institutions to determine the degree of transparency of information and voluntary compliance with capital market requirements. [42]. Organizing Investor Relations (IR) in a modern economy [43] must be a constant concern of companies in order to reduce the risks that may arise from the communication and presentation of financial and non-financial information. Among the principles of corporate governance is the existence of an investor relations department, so Ernst and Young [44] conducted a survey in 2018 with the participation of 876 professionals in the field of IR. The conclusions of the survey referred to the need to build strong relationships, the two-way communication between investors and management, the need for a strategy and a manual on IR, a close link between the IR department and the other departments and the fact that the IR department must be developed according to the size of the company.

The efficiency of the investor relations was the objective of several studies, that were conducted on their measurement and evaluation based on various indices developed by capital market institutions or correlations with various factors based on questionnaires, so Laskin and Laskin [45] proposed a mixed model of quantitative and qualitative methods. Karolyi et al. [46] used an IR index based on the answers to a questionnaire given by the staff of the IR departments of the international companies focused on the determinants of the IR organization.

Investor relations in companies listed at BVB have been analyzed in several previous researches, Albu and Girbina [47] analyzed a sample of companies that published complete data for 2010 and 2011 and concluded that there were a small number of companies that provided information on corporate governance, but still growing from year after year. Apostol [48] found that in the analyzed period 2011–2013, the number of companies that implemented the principles of corporate governance increased from year to year. Rogoz [49] analyzed the same topic for the financial investment companies based on data published in 2016 and identified an average degree of implementation of the principles of 88%. Bogdan and Dumitrescu [50] concluded that in 2018, the listed companies had a high degree of compliance with the principles of corporate governance, realizing that good corporate governance can ensure the sustainable development of the company.

In Romania, BVB collaborated with the Romanian Investor Relations Association (ARIR) to evaluate the practices of communication with investors. ARIR created the indicator of communication with investors for listed companies, called Vektor, [51] based on a methodology for assessing investor relations, in which 15 criteria were selected, grouped into six categories, giving each criterion a maximum score. The highest shares are held by promoting pro and interactive practices in the relationship with investors (60%), followed by the criteria regarding the existence of an investor relations office and the presentation of information related to corporate governance (30%), and a lower share the criteria for financial analysis and non-financial reporting were given (10%). The Vektor indicator is unique in Europe [52] and becomes an important source of data in research, being used in studies on the quality and transparency of financial reporting [53,54].

## 3. Materials and Methods

In order to achieve the objective of the paper, a quantitative and qualitative analysis was conducted based on data from the IRIS platform of the BVB [55]. The aim of the platform is to increase the degree of transparency by implementing a secure and credible communication system that could permanently ensure access to information and improve communication with shareholders, analysts and investors.

The empirical research started from a previous study [56] which identified the factors that influence business communication and, in this paper, we developed the study with additional variables and analyzed them from a different perspective in COVID-19 context. From all the published reports posted on IRIS platform in the period 16 March–14 May 2020, of the 83 companies listed at BVB, we included in our study only the ones that contained the following keywords: COVID-19, pandemic, coronavirus. Other sources of data were represented by the websites of the companies, from which we manually collect the information regarding their activities during the selected period.

We chose as indicators for measuring the level of communication made by companies, the number of published reports, that referred to the incidence of COVID-19, and the implementation of a business continuity plan. Given that it was a period of maximum crisis, we considered that the degree of communication of companies can be expressed by the number of published reports. Regarding the content of the reports, a qualitative analysis will be made. The companies that published public reports (R), the number of published reports and their content was identified.

The number of reports published by companies only partially reflects the concern for effective communication with investors. It is also important the content of the reports, respectively the way in which the companies structured the information and presented the measures taken in order to reduce the effects of the pandemic. Thus, the publication of the business continuity plan with the possible scenarios can be considered a relevant indicator in assessing the companies’ communication.

In order to evaluate the quality of the communication, the information was correlated with the evaluation made by ARIR published in January 2020 [57]. The Vektor indicator is most relevant for the Romanian-listed companies because it highlights the level of communication with shareholders and stakeholders.

The analysis was correlated with the profitability of the companies, because we considered that the existence of profit influences the communication with shareholders in terms of dividends that could be approved. Another indicator that can influence communication is the donations made by companies for contributions to combat the COVID 19 pandemic, which positively influences stakeholder relations [25]. The communication with stakeholders is more intense in companies that have over 500 employees in terms of the obligation to prepare sustainability reports [58], and they are interested in having a closer relationship with investors to strengthen their confidence. Therefore, it was included in the econometric model.

The trading category of companies is the indicator that classifies companies according to the degree of liquidity on the stock market, so BVB divides companies into two categories: Premium—those with high liquidity and is among the top 25 companies traded—and Standard. In addition to these two categories, the companies from the International category, respectively companies that have their headquarters abroad and are listed at BVB were added [59]. This indicator might have a direct relationship with the level of communication with both investors and stakeholders.

The form of ownership of the company can influence the way it is managed, this being different for private companies than for state-owned ones [60]. Private property can be owned by individuals, investment funds or employees’ associations. Therefore, if governance is different than communication strategies may also differ.

The description of the analyzed indicators is presented in Table 1.

Based on the selected indicators, an econometric model was constructed on a sample of 78 companies. We start from the maximum number of 83 companies, but 5 of them were eliminated, respectively those that were insolvent as well as those that were not evaluated by ARIR. Based on the data and characteristics of the companies presented above, the most relevant variables that support our objective were tested. For the first model, we used a panel data regression analysis in which we consider as dependent variable the number of published reports (R) and as independent variables: Vektor-communication evaluation indicator (V), profitability of the companies (P) and donations reports (D). To control the reliability of the model and reduce the risk of biases it was introduced in the regression the number of employees (E), the category of the company (C) and the type of shareholders (S) as a control variable. The form of the regression model is as follows:

R = α_it_ + β_1_V + β_2_P + β_3_D + β_4_E + β_5_C + β_6_S + ε_it_. (1)

To validate the econometric model, we tested for multivariate normality using Doornik–Hansen test and for symmetric correlation using Lawley test. To find the probability distribution and parameters that best describe the observed data we decided to use the maximum likelihood estimation within a random-effects regression. This type of regression was often used on COVID-19 related studies [61,62], due to the recent appearance of the pandemic and the short period of time available to analyze the data.

For the second model, we used a logistic regression in which we consider the Business continuity plan (B) as the dependent variable and the independent variables the same as in the previous model. This type of regression is used when the dependent variable is a dummy one and it was also used by other researchers [63] to study the impact of the pandemic on the stock market companies. The form of the regression is as follows:

B = α_it_ + β_1_V + β_2_P + β_3_D + β_4_E + β_5_C + β_6_S + ε_it_. (2)

All the tests and estimations were done in Stata Statistical Software: Release 13 (StataCorp LP, College Station, TX, USA).

## 4. Results and Discussion

The empirical analysis began with the study of the information regarding the companies and whether they published reports based on the selected keywords.

In order to highlight the level of communication of companies, Table 2 presents the number of public reports made by each company correlated with their classification base on the Vektor indicator.

In Table 2 it can be observed that 80% of the companies were in the category of those who had good communication, and 62% of them published at least a single report. Among the companies with less appreciated communication (<5), it is noteworthy that only 13% published more than one report. From the companies that were not included in Vektor indicator, only one published a single report and the other four had no communication.

**Figure 1:**
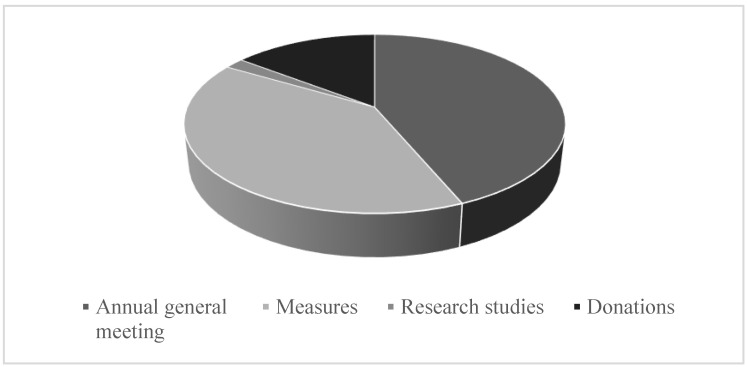
The structure of public reports. Source: own computation.

In Figure 1 is a graphic representation of the content of public reports regarding COVID-19 that included various information, such as shareholders meeting (44%), measures taken by companies (40%), research studies on coronavirus (2%) and donations (15%).

Considering that the analyzed period overlapped with the period in which the annual general meetings of shareholders were convened, it is understandable that the reports regarding the convocations had a more significant weight.

Current reports of measures taken by companies as part of the risk management to prevent coronavirus infection and limit its spread have focused on three areas: employees protection, customers protection, and business continuity plan. The analysis of the reports communicated by the companies showed that the most important measures announced by the companies, to deal with the crisis situation, were: providing protective equipment to employees; their ability to work from home; limiting the physical access of customers and ensuring the conditions of online relationships with them; as well as ensuring the supply chain and forecasting the evolution of the business. The four listed companies in the pharmaceutical sector have adapted their production to meet the needs of the medical system.

The public reports on the research studies conducted on COVID-19 were made by two of the companies listed on the stock exchange operating in the field of pharmaceutical manufacturing and in the field of healthcare (Antibiotice S.A. and Medlife S.A.). Antibiotice S.A. has resumed the emergency production of the drugs Paracetamol and Metamizole sodium because they have been included in most national treatment guidelines in the European Union (EU), as the first option to initiate the treatment of fever or pain caused by COVID-19 infection. Medlife conducted a study on the natural immunization of the population for COVID-19 and found that the population does not have naturally acquired immunity to COVID 19 [64].

The donations made by companies listed on the stock exchange were the subject of public reports and consisted in financing the acquisition of protective materials and medical equipment for hospitals in an estimated total amount of 7 million euros [65].

The frequency of the number of public reports of each company and their content is presented in Table 3.

The previous table shows that more than half of the companies that published a single report (58%) referred to the annual general meeting, followed by the reports announcing the measures taken by them (34%). In the case of companies that published two reports, their content was mainly related to the same topics mentioned above.

Table 4 presents an overview of the studied variables, grouped on three types based on Vektor indicator (with a score higher than 5, lower than 5 and not evaluated). There are 24 companies with a score higher than 5 and an average index of 8.08 and all of them published reports. Almost all of them (23) registered profit and half of them made donations. Regarding the number of employees, 15 of the companies have more than 500 and 17 of them are in the Premium trading category.

Two-thirds of the companies (54) were evaluated with a score lower than 5, and only 41 of them published reports. Regarding the profitability, 42 companies registered profit and only 3 made donations. 13 companies have more than 500 employees and only 8 are in the Premium trading category.

In the third group are the companies that were not evaluated in Vektor indicator. From the total of five, four of them are in insolvency, only one company published reports and none made donations. Three of the companies have more than 500 employees and only one is in the International trading category.

As it is shown in the table above, 37% of the total number of companies had over 500 employees in 2019, half of them have a Vektor indicator higher than 5 points and 87% had communications published during the analyzed period. Companies with less than 500 employees have a lower degree of communication, as only 17% have a Vektor score of more than 5 and 75% have published reports addressed to investors.

Most companies published their public reports (80%) and all of them were in the Premium trading category. The other companies had a weaker communication, two of the three International trading companies published reports and from the Standard ones only 70%. The Standard category also includes companies that are in insolvency procedure (4 companies) and this would be one of the reasons why they did not make a public report, as well as companies that are less traded on the stock exchange.

There are differences between the management and the administration of the business depending on the behavior or the shareholders, because the shareholders are the ones who make decisions in the companies.

Table 5 shows that 76% of the companies are owned by individuals and private companies, 11% are owned by financial investment companies, 10% are owned by the state and only 3% are owned by employees’ associations following privatization process. Comparing the form of ownership with the Vektor value, 75% of state-owned companies had better ratings (over 5 points) compared to 30% of private companies. The other two forms owned by investment companies and owned by employees’ associations registered below 5 points of Vektor value. One reason for these results can be the fact that the decision-making power is very concentrated in the first two types of ownership and they did not communicate publicly with the majority of shareholders or other interested parties. All categories had a good communication only 27% from the privately-owned shareholders did not published reports.

For the econometric analysis from the total of 83 companies, we excluded 5 because they weren’t included in the Vektor evaluation. The descriptive statistics, based on 78 companies, of the analyzed indicators are presented in Table 6. The dependent variables from the two models are Public Reports, which show that the companies had in average 1.31 communications and respectively, Business continuity plan, which contains the measures to assure the continuity of the business, was published by 46% of the companies. The indicator Vektor had a low value on average 3.72, which is due to the fact that a large number of companies did not obtain a score greater than or equal to 5. In average, 83% of the companies registered profit in 2019 but only 19% of companies reported donations and 36% of the companies had more than 500 employees. Regarding the profile of the companies, in average 35% of them are included in the premium and international trading category. Considering the form of the ownership, the largest share is held by the private sector, confirmed also by the mean of 2.72, but also due to the fact that the state is the majority shareholder in only 10% of the analyzed companies.

The correlations between the four variables analyzed are presented in Table 7, which shows that the first dependent variable Reports (R) is in a strong positive correlation (at 1% significance level) with the Vektor indicator (V) (0.62), with Donations (D) (0.57), Trading category (C) (0.42) and Number of employees (E) (0.29). The second dependent variable Business continuity Plan (B) is in a positive correlation (at 1% significance level) with Vektor indicator (V) (0.50) and Number of employees (E) (0.32).

Among the independent variables, the most relevant positive correlations identified are between Vektor (V) and the other 2 variables: Donations (D) (0.53) and Trading category (C) (0.65). A negative relation seems to exist between the independent variable Shareholders (S) and all the other variables, including the dependent one. This can be explained by the fact that all State-owned companies had intensive communication throughout published reports, compared to the privately owned companies.

Before validating the model, we tested the variables for multivariate normality using Doornik–Hansen test and for symmetric correlation using Lawley test. The results of the test rejected the null hypothesis of multivariate normality (Chi2(14) = 392.783 and Prob > chi2 = 0.0000) and also the hypothesis that the correlation matrix is compound symmetric (Chi2(20) = 121.41 and Prob > chi2 = 0.0000) and conclude that there are probably differences in the correlations of variables.

Performing a simple linear regression, we obtained an R-squared of 0.45, which means that there is a medium significant link between the variables, respectively the modification of the independent variables influences in a proportion of 45% the modification of the dependent variable. F (6, 65) is 9.05 at 1% level of significance, higher than the critical level, concluding that the model is valid.

Next, we used a likelihood estimation random-effects regression to show the influence of each independent variable. The results can be seen in Table 8, the coefficients of correlation together with t values from the Student test in parentheses and the significance level.

The value obtained for Wald chi2 was bigger than the threshold at 1% level of confidence, which shows that the model is statistically significant. Two of the control variables, number of employees and the trading category of the companies, were not validated, not even at a 10% significance level. Donations have the biggest influence on reporting, at a 99% statistical confidence level, if the companies report the donations, the number of public reports may increase by 79%. If the companies register profit, at a 90% statistic confidence level, the numbers of public reports might increase by 31%. As for the communication evaluation indicator (Vektor), if it increases by one level, at 99% confidence level, it might lead to an increase in public reporting by 12%.

For the second model, we obtained a Wald chi2 value bigger than the threshold at 1% level of confidence, therefore this model is also statistically significant but only two variables were validated at 1% statistically significance, Vektor and Shareholders. Considering the odds ratio obtained from the logistic regression, resulted in the odds that the companies made a business continuity plan being increased by 0.46% if they registered a higher score at Vektor indicator and decreased by 0.61% if the companies had a majority of private shareholders.

The impact of the published reports on the shareholders is highlighted in the way the annual general meeting was organized, because all these took place through online voting, which involved the adoption of urgent procedures for holding meetings in good conditions. This aspect has a legal character which implies the change of the constitutive acts of the companies, as well as implications at the legislative level [38,39]. Similar results were obtained by Atkins et al. [25] in their study of COVID-19 impact announcements, published by companies listed on the Johannesburg Stock Exchange from South Africa.

Another consequence of the COVID-19 pandemic was the fluctuations of the stock prices listed at BVB, which correlated with the evolution of international markets. At the date of approval of the state of emergency, the stock market fell sharply, but by the end of the state of emergency, a part of the loss was recovered [66,67].

The published reports had an impact on the stakeholders, by the fact that they became aware of the measures taken by companies, of the donations made and were encouraged to donate or participate together with companies in humanitarian campaigns actions. This implicitly led to the consolidation of the companies’ prestige in the community. We exemplify the initiative of Banca Transilvania, whose shares were the most traded in the analyzed period, which manifested itself through the public support of charitable actions meant to collect funds and resources to support medical units in their fight against the COVID-19 pandemic [68].

Based on the analyzed data, it can be summarized that most Romanian-listed companies were concerned to publish as many public reports as possible in the context of the pandemic and that they quickly adapted to the crisis situation created, and the companies’ activity was not interrupted. Of course, the pandemic affected the performance of companies in the short term, but a large share of companies listed at BVB have been profitable in the previous years, which will help them cover possible losses in 2020. Good communication with all those involved can have an impact on the reputation of the companies, as well as on their profitability, as can be seen from the Vektor indicator.

The proposed econometric model showed that there is a direct relationship between the number of public reports and the communication evaluation indicator, profitability of companies and the announcement of the donations made. The results of the research were confirmed in other research, which showed that good communication contributes to increasing the reputation of companies and implicitly the company’s evaluation index [35,69].

The level of communication measured by the degree of transparency of financial and non-financial information is correlated with previous research based on listed companies in Romania [47,48,49,50] structured on the analyzed categories. Thus, we found a positive evolution of the concerns of companies in all fields of activity to communicate more information, in special the companies from the pharmaceutical industry [54].

The quality of governance is influenced by a number of factors, at both microeconomic [70] and macroeconomic level [71]. Economic crises have a direct link between governance and economic growth, which leads to the need for long-term strategies to have good governance [72,73,74]. Thus, the managers of listed companies should be concerned to improve the communication, considering the evaluation criteria within the Vektor indicator evaluation methodology, especially the improvement of aspects related to corporate governance disclosure and proactive approach to investor relations. The organization of an investor relations office has the role of being an interface with the capital markets, investors, shareholders and groups of analysts, so as to understand the current and future activity of the company.

## 5. Conclusions

The global pandemic has brought many challenges to corporate governance, being subject to unprecedented medical restrictions, and requiring rapid adaptation of production strategies and a rethinking of product and material flows to meet staff protection requirements, but also of the community in which it operates. The actions of the leaders have changed radically, by amplifying the implementation of responsible measures to ensure care for the other members of the organization but also for all members of society, represented by the community. In this condition, the investor relations and communication reports of the activity of listed companies are very important to maintain their confidence in the community [74].

Through this paper were presented the theoretical and practical aspects related to corporate communication during the state of emergency caused by COVID-19 of the companies listed at BVB. The study showed that in most cases, the companies had at least one public report, especially the one related to the annual shareholders meeting, a percentage of 14% companies had two public reports, and only 13% of companies have published three or more reports COVID-19 related. The content of the public reports regarding COVID-19 included various information, such as shareholders meeting, measures taken by companies, research studies on coronavirus and donations made by companies.

The results from the econometric models showed that if the studied companies registered profit the numbers of public reports might increase by 31% and if Vektor indicator increased by one level it might lead to an increase in public reporting by 12%. The odds that the companies implemented a business continuity plan increased by 0.46% if they registered a higher score at Vektor indicator and the odds decreased by 0.61% if the companies had a majority of private shareholders.

The content of the public reports regarding the business continuity plan helps the stakeholders to understand the measures that were implemented for the safety of the employees and the communication with the suppliers and customers.

In the context studied in this paper, we highlighted the responsible communication of Romanian companies that manifested through leadership decisions to protect the employees, the business and the community in which they operate. This way the companies became aware of the effects of the pandemic, and socially responsible for compliance of safety by implementing social distancing measures. The taken measures were designed to protect each person and the group he belongs to while generating safety and protection for the whole family. These measures are in the best interest of both the person and the organization, which will implicitly increase the confidence in the company, as a factor of stability within the community.

We conclude that this pandemic crisis has generated an extension of social responsibility to people within an organization, which joins its efforts to ensure public health, by combating the effects of the pandemic and protect the entire community, which has an impact on supporting the economy, affected by restrictive measures imposed by the state authority to all companies.

The originality of this research is based on the use of Vektor indicator in the analysis, which only a small number of papers from Romanian literature employed [53,54], and also on the fact that the research of risk management associated with COVID-19 is a new and growing one with many opportunities to be developed.

This article contributes to the recent literature on the impact of COVID-19 on business communication and finance. Starting from the concept of social responsibility of organization defined by Manuel Velasquez [13] as that action in which participants cooperate to achieve the proposed objectives [10], we can state that, in the same way, the organizations will act and cooperate both with each other, and with the state or community institutions, actively involved in combating the effects of the global pandemic crisis. The paper can be a bibliographic source for researchers in the field of risk management associated with COVID-19 and corporate governance and may have practical implications for managers by resetting priorities by focusing on both the organizational and social spheres, in order to respond promptly to all the challenges posed by the pandemic crisis.

The limits of the research can be considered to be the manually collected data, which implies a certain risk of bias and the short period of time for analysis, but we considered it relevant in the pandemic context. Another limitation is that the study was conducted on a single financial market. Future directions of research can be oriented to the impact and the effects of the pandemic in the short and long term on financial and non-financial indicators of listed companies and can be extended with comparative analyzes on several financial markets.

## Figures and Tables

**Table 1:** Description of the variables.

Variables	Description
Public Reports (R)	0 to 4 according to Table no 2
Business Continuity Plan (B)	1–if the company had public reports 0–if the company didn’t have public reports
Vektor (V)	0 to 10 according to the ARIR methodology
Profitability (P)	1–if the company recorded profit 0–if the company recorded losses
Donations (D)	1–if the company reported donations 0–if the company did not reported donations
Employees (E)	1–if the company has more than 500 employees 0–if the company has less than 500 employees
Category (C)	1–premium or international trading category 0–standard trading category
Shareholders (S)	1–if the majority shareholder is the state 2–if the majority shareholder is an investment company 3–if the majority shareholder is private (individuals and private companies) 4–if the majority shareholder is the employees’ association

Source: Authors’ own work.

**Table 2:** The number of public reports communicated.

No. of Public Reports	No. of Companies	Vektor		
		≥5	<5	None
0	17	0	13	4
1	41	6	34	1
2	14	9	5	0
3	10	9	1	0
4	1	0	1	0
Total	83	24	54	5

Source: own computation using IRIS platform data.

**Table 3:** The content of the public reports.

No. of R	Annual General Meetings	Business Continuity Plan	Research Studies	Donations	Total
1	24	14	0	3	41
2	12	12	0	4	28
3	9	12	2	7	30
4	0	3	0	1	4
Total	45	41	2	15	103

Source: own computation using IRIS platform data.

**Table 4:** Variables overview.

Vektor Index		No. of Comp	Public Reports		Profitability		Donation		No. of Employees		Trading Category		
Value	Aver.		Yes	No	Yes	No	Yes	No	<500	≥500	Pr	Int	St
≥5	8.08	24	24	0	23	1	12	12	9	15	17	2	5
<5	1.82	54	41	13	42	12	3	51	41	13	8	0	46
None		5	1	4	2	3	0	5	2	3	0	1	4
Total	3.72	83	66	17	67	16	15	68	52	31	25	3	55

Source: own computation using IRIS platform and BVB data.

**Table 5:** Forms of ownership of the majority shareholder.

Shareholders	Code	No. of Companies	Published Reports		Vektor Value		
			Yes	No	≥5	<5	None
State	1	8	8	0	6	2	0
Investment companies	2	9	9	0	1	8	0
Private	3	63	46	17	17	41	5
Employees’ associations	4	3	3	0	0	3	0
Total		83	66	17	24	54	5

Source: own computation using IRIS platform and BVB data.

**Table 6:** Descriptive statistics.

Variables	Obs.	Mean	Std. Dev.
R	78	1.3077	0.9440
B	78	0.4615	0.5017
V	78	3.7244	3.2660
P	78	0.8333	0.3751
D	78	0.1923	0.3967
E	78	0.3589	0.4828
C	78	0.3461	0.4788
S	78	2.7179	0.7006

Source: own computation using Stata 13.

**Table 7:** Correlation matrix.

	R	B	V	P	D	E	C	S
R	1							
B	0.5737 ***	1						
V	0.6239 ***	0.506 ***	1					
P	0.2201 *	0.138	0.2642 *	1				
D	0.5683 ***	0.200 *	0.5327 ***	0.1309	1			
E	0.2959 ***	0.325 ***	0.3683 ***	0.0478	0.2452 *	1		
C	0.4221 ***	0.245 **	0.6556 ***	0.2531 *	0.3971 ***	0.1858	1	
S	−0.2205 **	−0.216 **	−0.2274 **	−0.0824	−0.2229 **	−0.2727	−0.1697	1

*—10% level of significance, **—5% level of significance, ***—1% level of significance, Source: own computation using Stata 13.

**Table 8:** Results of regression.

D.V.	Obs.	Log-Likelihood	Wald Chi2	V	P	D	E	C	S
R	78	−81.948	343.99 ***	0.1263 (3.47) ***	0.3185 (1.60) *	0.794 (3.35) ***	0.184 (1.06)	−0.022 (−0.10)	0.123 (2.16) **
B	78	−40.511	18.41 ***	0.463 (3.14) ***	0.059 (0.09)	−0.742 (−0.81)	0.649 (1.09)	−0.850 (−1.01)	−0.615 (−2.72) ***

*—10% level of significance, **—5% level of significance, ***—1% level of significance, Source: own computation using Stata 13.
